# The Prevalence of Hepatitis C Virus (HCV) among Lichen Planus Patients and Its Clinical Pattern at the University of Abuja Teaching Hospital (U.A.T.H), Gwagwalada, Abuja, Nigeria

**DOI:** 10.5539/gjhs.v4n5p113

**Published:** 2012-08-06

**Authors:** Ukonu Agwu Bob, Uhunmwangho Augustine

**Affiliations:** 1 University of Abuja Teaching Hospital, Gwagwalada, Abuja, Nigeria

**Keywords:** hepatitis C virus, lichen planus, epidemiology

## Abstract

**Objective:**

The relationship between hepatitis C virus and Lichen Planus have been widely reported in the literature; although there are wide geographical variations in the reported prevalence of hepatitis C virus infection in patients with lichen planus. This study seeks to determine the prevalence of hepatitis C virus among lichen planus patients and its clinical morphological type in the University of Abuja Teaching Hospital, Gwagwalada Abuja, Nigeria.

**Materials/Methods:**

This study was conducted between January 2010 and December, 2011 at the out patients Dermatological unit of the department of medicine at the University of Abuja Teaching Hospital Gwagwalada Abuja, Nigeria. Consecutive patients who had body eruptions suspected to be lichen planus were recruited and histology done for confirmation. The control group included patients’ relations and some dermatology patients known to have low risk of hepatitis C virus infection and liver function tests done for both subjects and control after obtaining oral consent from them to participate in the study.

**Result:**

Anti- HCV antibodies were detected in nine cases (21.4%) and one case (3.3%) in the control group. This was statistically significant difference between the HCV antibody among the subject and control group (P<0.038). Hypertrophic lichen planus was the most frequent clinical type. Liver function test was not statistically significant among the subject and control group.

**Conclusion:**

Lichen planus and Hepatitis C virus appear to have a relationship and the prevalence rate was higher among the subject as compared to the control group in our environment.

## 1. Introduction

Lichen planus is an inflammatory mucocutaneous condition that usually exhibits a distinctive pattern or morphology. It is likely that both endogenous genetic and exogenous environmental components such as drugs and/or some infection(s) may interact to elicit the disease (Boyd & Nelder, 1991; Daoud et al., 1999). An increased prevalence of chronic liver disease has been reported in patients with lichen planus; this includes biliary cirrhosis and chronic viral infection like hepatitis C virus which has been implicated in triggering lichen planus (Mokni et al., 1991; Sanchez-perez et al., 1996; Chuang et al., 1999).

There has been an increase in the prevalence of lichen planus (especially oral and erosive lichen planus) and hepatitis C virus with the discovery of hepatitis C virus, a single stranded RNA virus in 1989 and the availability of test for antibodies in1991. Various studies conducted in different parts of the world have proved or disproved a causative role for hepatitis C virus in lichen planus (del Olmo et al., 1999; Wagoo, 2000; Migogna, 2000; Criber, 1994).

This study was conducted to determine whether there was a statistically significant relationship between hepatitis C virus in patients with lichen planus in the University of Abuja Teaching Hospital, Abuja Nigeria.

## 2. Materials/Methods

The study was conducted between January 2010 and December 2011 during which a total number of seven hundred and twenty (720) new patients were seen on the outpatient Dermatology Unit of the University of Abuja Teaching Hospital, Abuja. Forty-two (42) were newly diagnosed cases of lichen planus, which accounts for 5.8% of the total cases seen during the period. There were 24 (57.1%) male and 18 (42.9%) female. Diagnosis was based on clinical observation and histology where necessary. Patients who were on anti-hypertensive like bêta-blockers, thiazide diuretics or non steroidal anti-inflammatory agents before noticing the eruptions were excluded from the study.

The control group included patients’ relations and some dermatological patients who had dermatological cases like acne vulgaris known to have low risk for hepatitis C virus infection. The subjects and the control group were screened for the presence of anti- hepatitis C virus antibodies by the third generation ELISA and liver function tests after obtaining oral consent from them to participate in the study. The data generated were keyed into SPSS 16.0 for statistical analysis. Statistical tools employed were Student t-test, frequency, Fisher’s exact test, chi-square, crosstabs’ statistics and univarite analysis.

## 3. Results

Nine 9 (21.4%) of 42 subject group had positive titre for anti- HCV antibody while one 1 (3.3%) of the control group had positive titre for anti-HCV anti-body. P = 0.038 shows a statistically significant relationship between hepatitis C virus and lichen planus. Relative risk ratio (0.156) (RR) < 1 for those who had positive anti- HCV antibodies indicates that those who were HCV positive were more susceptible to having lichen planus. Relative risk ratio (0.591) (RR) < 1 for those who had lichen planus indicates that, those who had lichen planus were more vulnerable to having HCV anti-bodies.

The details of the bio-data of both the subjects and the control groups are seen in table 1. Age Range for subject group 12-68, mean age = 36.76 years; P-value 0.999 and 0.972 show that there was age and sex match respectively among the Subject. Age Range for control group 18-50, mean Age = 34.43years; P-value of 0.999 and 0.985 show that there was age and sex match respectively among the control group. The ratio of male to female was 1.33:1.

**Table 1:** The relationship between HCV status of the subject and control group’s bio data

	Subject									Control				
:	HCV –ve Freq (%)		HCV +ve Freq (%)		Total Freq (%)		P-Value	HCV -ve Freq (%)		HCV +ve Freq (%)		Total Freq (%)		P-Value
**SEX**													
Male	16	(38.1)	8	(19.0)	24	(57.1)	0.102	15	(50)	1	(3.3)	16	(53.3)	
Female	17	(40.5)	1	(2.4)	18	(42.9)	0.000	14	(46.7)	0	(0)	14	(46.7)	
**Total**	**33**	**(78.6)**	**9**	**(21.4)**	**42**	**(100)**	**0.033**	**29**	**(96.7)**	**1**	**(3.3)**	**30**	**(100)**	**1.000**
**EDUCATION**:													
No Education	2	(4.8)	0	(0)	2	(4.8)	-	1	(3.3)	0	(0)	1	(3.3)	
Primary Education	6	(14.3)	3	(7.1)	9	(21.4)	0.317	6	(20.0)	0	(0)	6	(20.0)	
Secondary Education	11	(26.2)	4	(9.5)	15	(35.7)	0.071	9	(30.0)	1	(3.3)	10	(33.3)	
Tertiary Education	14	(33.3)	2	(4.8)	16	(38.1)		13	(43.3)	0	(0)	13	(43.3)	
**Total**	**33**	**(78.6)**	**9**	**(21.4)**	**42**	**(100)**	**0.512**	**29**	**(96.7)**	**1**	**(3.3)**	**30**	**(100)**	**0.558**
**OCCUPATION**:														
Retired Civil Servant	1	(2.4)	0	(0)	2	(4.8)		-		-		-		
Civil Servant	4	(9.5)	2	(4.8)	6	(14.4)		6	(20)	0	(0)	6	(20)	
Security Job	0	(0)	2	(4.8)	2	(4.8)		-		-		-		
Police/Military/Paramilitary	0	(0)	1	(2.4)	1	(0)		-		-		-		
Trader	1	(2.4)	0	(0)	1	(2.4)		-		-		-		
Student	10	(23.8)	0	(0)	10	(23.8)		8	(26.7)	0	(0)	8	(26.7)	
House Wife	8	(19.0)	0	(0)	8	(19.0)		4	(13.3)	0	(0)	4	(13.3)	
Business	2	(4.8)	3	(7.1)	5	(11.9)		3	(10.0)	0	(0)	3	(10.0)	
Farmer	3	(7.1)	1	(2.4)	4	(9.5)		2	(6.7)	0	(0)	2	(6.7)	
Teacher	2	(4.8)	0	(0)	2	(4.8)		3	(10.0)	0	(0)	3	(10.0)	
Applicant	2	(4.8)	0	(0)	2	(4.8)		1	(3.3)	0	(0)	1	(3.3)	
Journalist	-		-		-			(3.3)	1	0	(0)	1	(3.3)	
Artisan	-		-		-			1	(3.3)	0	(0)	1	(3.3)	
Hotelier	-		-		-			0	(0)	1	(3.3)	1	(3.3)	
TOTAL	33	(78.6)	9	(21.4)	42	(100)	0.013	29	(96.7)	1	(3.3)	30	(100)	

Table 2 shows the relationship between clinical type of lichen planus and hepatitis C virus infection anti bodies. Hypertrophic clinical type was the most common at frequency of 21 (54.3%). Appendages and mucosal involvement shows nail 1 (2.4%), genital 2 (4.8%) and scalp 1 (2.4%) respectively. Only one of the lichen planus with oral mucosal involvement was HCV antibody positive.

**Table 2:** The relationship between hepatitis C status of subject and the clinical diagnosis and other mucosal involvement respectively

Clinical Type	HCV -ve Freq (%)		HCV -ve Freq (%)		Total Freq (%)		P-Value	Mucosal /appendages Involvement	HCV -ve Freq (%)		HCV +ve Freq (%)		Total Freq (%)	
Annular	4	(9.5)	0	(0)	4	(9.5)		None	29	(69.0)	7	(16.7)	36	(85.7)
Follicular	2	(4.8)	0	(0)	2	(4.8)		Nail	0	(0)	1	(2.4)	1	(2.4)
Hypertrophic	15	(35.7)	6	(28.6)	21	(54.3)		Genital	2	(4.8)	0	(0)	2	(4.8)
Annular& Follicular	4	(9.5)	0	(0)	4	(9.5)		Oral-			
Hypertrophic & Annular	2	(4.8)	2	(4.8)	4	(9.5)		Mucosal	1	(2.4)	1	(2.4)	2	(4.8)
Ulcerative	0	(0)	1	(2.4)	1	(2.4)		Scalp	1	(2.4)	0	(0)	1	(0)
Linear Annular	2	(4.8)	0	(0)	2	(4.8)		**Total**	**33**	**(78.6)**	**9**	**(21.4)**	**42**	**(100)**
Linear Hypertrophic	1	(2.4)	0	(0)	1	(2.4)		**P-Value**					**0.236**	
Bullous	1	(2.4)	0	(0)	1	(2.4)								
Atrophic	1	(2.4)	0	(0)	1	(2.4)								
Hypertrophic & Follicular	1	(2.4)	0	(0)	1	(2.4)								
**Total**	**33**	**(78.6)**	**9**	**(21.4)**	**42**	**(100)**	**0.389**							

In seven (7) (16.7%) of the subjects had total bilirubin higher than normal range while thirteen (13) (30.9%) of the patients had higher than normal Transaminase and eleven (11) (26.2%) had normal Aspartate Transaminase. Eta = 0.807 shows a high degree of association between those that are HCV positive/negative with lichen planus and total bilirubin/ total liver function test (P- Value = 0.896 and R = -0.014), although it was not statistically significant. Eta= 0.619 shows a high degree of association between those that are HCV positive/negative with lichen planus and Aspartate Transaminase (R^2^ = 0.190; P-value = 0.187) but it was not statistically significant. Eta = 0.738 shows a high degree of association between those that are HCV positive/negative with lichen planus and Alanine Transaminase (R^2^ = 0.255; P-value = 0.044) and this was statistically significant {R^2^ = Kendall’s tau-b correlation}. Among those without lichen planus, there was no statistical difference in their liver function test/total bilirubin, Alanine Transaminase, and Aspartate Transaminase P = 0.994, 1.000, 1.000 respectively. The Mean duration of disease was 14.12months, range 6-24months.

## 4. Discussion

There appears to be wide geographical variations in the proposed relationship between lichen planus and chronic hepatitis C virus infection. In a study from England (Tucker, 1999) and India (Prabhu, 2002) there seems to be no relationship. However, a study conducted in Japan shows that 37.8% of 45 patients with lichen planus had serological evidence of hepatitis C virus infection (Tanei, 2002). Also, in Nigeria, anti HCV antibodies were found in 9% of the patients with lichen planus (Daramola et al., 2002). Surprisingly, Italy, Spain and Germany were other countries in which high levels of HCV infection had been described among patients with lichen planus (Schanez-perez et al., 1996; Foti et al., 1994; Inhof, 1997). Erkek et al. (2001) found that HCV antibodies prevalence in Turkish patients with lichen planus (12.9%) was higher than that of the control group (3.7%) but the difference was not statistically significant; whereas Kirtak et al 2000 found a statistically significant difference in Gaziantep region of Turkey. Daramola et al. 2002 in south west Nigeria found a 9% prevalence of HCV among 57 patients with lichen planus and there was no statistically significant association with HCV infection.

However, from our study, the prevalence of HCV infection in patients with lichen planus in Gwagwalada- Abuja, Nigeria was (21.4%). It was statistically significant and higher than the Anti HCV antibodies in the control group (3.3%) at P < 0.038.

Hypertrophic clinical type of lichen planus was found to be the most common and this is similar to previous observation (Daramola et al., 2002). Of interest was the fact that only one of the lichen planus patients had oral mucosa involvement and was found to be HCV positive. In other studies (Dupin et al., 1997; Carrozzo et al., 1996), mucosal erosions were more common in HCV patients (P < 0.001).

We also observed that seven (7) (16.7%) of the patients had total bilirubin higher than normal (>17µmol/L) but none of them were HCV positive. However, there was no statistically significant difference in the liver function test, which includes total bilirubin, aspartate transaminase and alanine transaminase of the subject. Overall, it was observed that there was no statistical association between the liver function test of those with HCV infection and those without HCV infection and this was in keeping with similar study (Tucker et al., 1999).

The role of HCV infection and the development of lichen planus is still indistinct. An immune-mediated mechanism involving activated T-cells, particularly CD+8 T-cells, directed against basal keratinocytes has been proposed (Hus) (Hussein, 2007). Up-regulation of intercellular adhesion molecule-1 (ICAM-1) and cytokines associated with Th 1 immune response, such as interferon (IFN)-gamma, tumour necrosis factor (TNF)- alpha, interleukin (IL)-1 alpha, IL-6 and IL-8, may also play a role in the pathogenesis of lichen planus (Chen et al., 2007).

The association of HCV infection and lichen planus is contentious therefore its pathogenic role is uncertain. According to some authors, the relationship between lichen planus and positive serology of HCV, even positive RNA is not substantive enough to determine the role of HCV in the pathogenesis of lichen planus. The demonstration of HCV RNA in epithelial cells of oral mucosa (Carrozzo et al., 1996; Arrieta et al., 2000) and skin lesions (Erkek et al., 2001) of patients with lichen planus would lead to the hypothesis that direct action of the virus is involved.

Hepatitis C Virus has been shown to be a potential antigen presented by Langerhans cells followed by activation and migration of lymphocytes resulting in damage to basal cells via cytokines of cytotoxic T-cells (Carrozzo et al., 1996, Nago, 1998). The virus may alter epithelial antigenicity at sites of mucocutaneous replication leading to either direct activation of cytotoxic T-cells (Nago et al., 1998; Pettuzzi, 2004) or production of antibodies against epithelial antigens (Lodi et al., 1997). Petruzzi et al. (2004) recently demonstrated differences in lymphocyte sub-populations between HCV positive oral lichen planus patients and HCV negative patients with oral lichen planus. They attributed this to the chronic antigenic stimulation of HCV. We presume that the different geographic endemicity levels of HCV provide adequate explanation for the conflicting results in different studies but factors such as host immune dysregulation or concomitant immunomodulatory infections might play a role.

## 5. Conclusion

In conclusion, though this study did not resolve the issue of pathogenic role of HCV infection in lichen planus but it shows a relationship between them, hence, there might be need to screen for anti HCV antibody in patients with lichen planus. Also, we are advocating that during emergencies where blood transfusion is urgently needed caution should be exercised when collecting blood from someone that has Lichen planus for blood transfusion. A multicentre study for a larger group of patient is needed to resolve this issue for a better policy formulation in our environment where most infections like hepatitis C virus is prevalent.

### Ethical Approval

An oral approval was gotten from the Head of Department of the Ethical committee in the University of Abuja Teaching Hospital Gwagwalada, Abuja before commencing the study.

**Figure:**
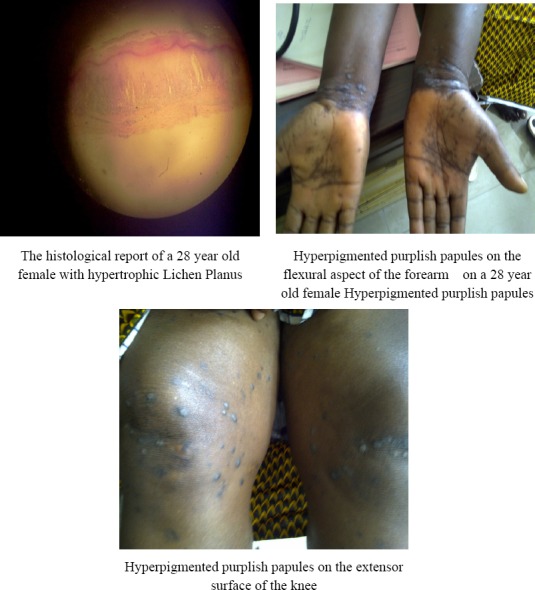
Clinical Pictures/histology of patients with Lichen Planus: Lichen Planus on a 28 year old female
